# Development of a Novel Vaccine Containing Binary Toxin for the Prevention of *Clostridium difficile* Disease with Enhanced Efficacy against NAP1 Strains

**DOI:** 10.1371/journal.pone.0170640

**Published:** 2017-01-26

**Authors:** Susan Secore, Su Wang, Julie Doughtry, Jinfu Xie, Matt Miezeiewski, Richard R. Rustandi, Melanie Horton, Rachel Xoconostle, Bei Wang, Catherine Lancaster, Adam Kristopeit, Sheng-Ching Wang, Sianny Christanti, Salvatore Vitelli, Marie-Pierre Gentile, Aaron Goerke, Julie Skinner, Erica Strable, David S. Thiriot, Jean-Luc Bodmer, Jon H. Heinrichs

**Affiliations:** 1 Vaccine Basic Research, Merck Research Laboratories, Merck and Company, Incorporated, West Point, Pennsylvania, United States of America; 2 Eurofins Laboratories, Lancaster, Pennsylvania, United States of America; 3 Vaccine Analytical Development, Merck Research Laboratories, Merck and Company, Incorporated, West Point, Pennsylvania, United States of America; 4 Vaccine Drug Product Development, Merck Research Laboratories, Merck and Company, Incorporated, West Point, Pennsylvania, United States of America; 5 Vaccine Process Development, Merck Research Laboratories, Merck and Company, Incorporated, West Point, Pennsylvania, United States of America; Cornell University, UNITED STATES

## Abstract

*Clostridium difficile* infections (CDI) are a leading cause of nosocomial diarrhea in the developed world. The main virulence factors of the bacterium are the large clostridial toxins (LCTs), TcdA and TcdB, which are largely responsible for the symptoms of the disease. Recent outbreaks of CDI have been associated with the emergence of hypervirulent strains, such as NAP1/BI/027, many strains of which also produce a third toxin, binary toxin (CDTa and CDTb). These hypervirulent strains have been associated with increased morbidity and higher mortality. Here we present pre-clinical data describing a novel tetravalent vaccine composed of attenuated forms of TcdA, TcdB and binary toxin components CDTa and CDTb. We demonstrate, using the Syrian golden hamster model of CDI, that the inclusion of binary toxin components CDTa and CDTb significantly improves the efficacy of the vaccine against challenge with NAP1 strains in comparison to vaccines containing only TcdA and TcdB antigens, while providing comparable efficacy against challenge with the prototypic, non-epidemic strain VPI10463. This combination vaccine elicits high neutralizing antibody titers against TcdA, TcdB and binary toxin in both hamsters and rhesus macaques. Finally we present data that binary toxin alone can act as a virulence factor in animal models. Taken together, these data strongly support the inclusion of binary toxin in a vaccine against CDI to provide enhanced protection from epidemic strains of *C*. *difficile*.

## Introduction

*Clostridium difficile* infections are the most widely recognized cause of hospital acquired infectious diarrhea [1]. There is a critical need for a vaccine for the prevention of this disease. A recent study by the Duke Infection Outreach Network found that *C*. *difficile* has superseded Methicillin-Resistant *Staphylococcus aureus* (MRSA) as the most common pathogen causing healthcare associated infections in the southeastern United States [2]. A recent bulletin from the Centers for Disease Control and Prevention (CDC) (http://www.cdc.gov/drugresistance/threat-report-2013) listed the current threat level from *C*. *difficile* as urgent. According to this CDC bulletin, there are 250,000 infections each year caused by this bacterium that require hospitalization or affect already hospitalized patients resulting in 14,000 deaths and at least $1 billion in excess medical costs each year. The organism is associated with persistent diarrhea primarily in individuals of advanced age with pre-existing co-morbidities, during prolonged hospitalization, and, most importantly, with the use of broad-spectrum antibiotics. Because the organism can form spores which are impervious to antibiotics, there is a significant risk of recurrence (about 30%).

Disease caused by *C*. *difficile* has primarily been attributed to the organism’s elaboration of the large clostridial toxins (LCTs): TcdA and TcdB. TcdA and TcdB glucosylate Rho-like GTPases leading to the depolymerization of filamentous actin (F-actin) of colonic epithelial cells, apoptosis and cell death. The disruption of the actin cytoskeleton leads to a loosening of the epithelial tight junctions resulting in excess fluid accumulation in the intestinal lumen. The severity of disease caused by *C*. *difficile* can range from mild diarrhea to fulminant pseudomembraneous colitis, and absent suitable treatment, toxic megacolon, and death.

Recently, epidemic strains of *C*. *difficile* have emerged and contributed to an increase in disease incidence, particularly in the United States and Canada [3, 4]. These strains are referred to as NAP1/BI/027, depending on the typing scheme used for characterization. NAP1 strains have been associated with more severe disease, greater frequency of recurrence, and increased mortality, although the association between strain type and enhanced disease is still debated [5]. Nevertheless, NAP1 strains possess many characteristics that have been postulated to be responsible for this increase in virulence. Among these are a deletion in the regulatory locus, *tcdC*, which has been associated with increased production of TcdA and TcdB, an increased rate of sporulation, and greater propensity for antibiotic resistance [3, 6–8]. Of particular interest is the expression of an additional toxin known as binary toxin.

Binary toxin is a member of the two component ADP-ribosylating toxin family, which also includes *Clostridium botulinum* C2 toxin, *Clostridium perfringens* iota toxin, *Clostridium spiroforme* toxin, and *Bacillus anthracis* edema and lethal toxins [9]. This toxin is composed of two separate components: CDTa, which is responsible for enzymatic activity, and CDTb, the binding component. CDTb is secreted by *C*. *difficile* as an inactive pro-protein and, in the duodenum, is activated through the proteolytic cleavage by chymotrypsin that allows CDTb to oligomerize and bind to its receptor. Next, CDTb binds to CDTa and facilitates its transport into the cytosol where CDTa ADP-ribosylates G-actin. This prevents actin polymerization and disrupts the actin cytoskeleton resulting in cell rounding and eventually cell death [10–12]. A recent study suggests, in addition to being cytotoxic, binary toxin may also play a role in bacterial adhesion [13]. In this report, binary toxin was shown to induce the formation of microtubules in epithelial cells. The authors theorized that these microtubules might provide an additional substrate for the binding of *C*. *difficile* leading to an increase in bacterial load in the gut of infected individuals. The exact role of binary toxin in disease pathology is still being investigated, however, evidence suggests that it plays only a minor role in disease pathology in animal models [14, 15].

Treatment of CDI has traditionally focused on discontinuation of the offending antibiotic treatment, followed by the use of antibiotics with higher specificity for the organism including vancomycin, metronidazole, and the recently approved, narrow-spectrum drug Fidaxomicin [16, 17]. However, these therapies can compound the problem by further disrupting the normal gut flora that serves as the natural defense against *C*. *difficile* colonization and infection. New approaches for the prevention and treatment of CDI are currently in development or being evaluated in clinical trials. Among these new approaches is a pair of monoclonal antibodies targeting TcdA and TcdB, which were recently evaluated in a phase III clinical trial and had previously been shown to reduce the rate of CDI recurrence [18]. In addition, several vaccines targeting TcdA and TcdB are currently in development [19–24] (for a recent review of both vaccine and antibody interventions see Heinrichs and Therien [25]). Here, we describe the development of a vaccine containing recombinant, attenuated TcdA and TcdB produced in insect cells using a baculovirus expression system. We demonstrate, using the Syrian golden hamster model, that this bivalent vaccine is highly efficacious against non-NAP1 strains but that it is not effective against strains that also produce binary toxin, despite the induction of potent neutralizing antibody responses to the LCTs. The addition of binary toxin antigens to this vaccine provided enhanced efficacy against NAP1 strains suggesting that protection for at-risk individuals exposed to these highly virulent strains is dependent on immune responses against both the LCTs and binary toxin.

## Methods

### Strains/ cell lines

*C*. *difficile* strain VPI10463 (TcdA+, TcdB+, CDT-, ribotype 087, ATCC#43255) was obtained from the American Type Culture Collection (ATCC, Manassas, VA). Strain BI17 (TcdA+, TcdB+, CDT+, ribotype 027) was generously provided by Dale Gerding (Hines VA Hospital, IL). Strain 8864 (TcdA-, TcdB+, CDT+, ribotype 036) was generously provided by Maja Rupnik (University of Ljubljana, Slovenia). Spores were prepared following established methods, aliquots were prepared in water, and frozen at -70°C for up to one year [26]. Spores were thawed prior to challenge and diluted in phosphate-buffered saline. *C*. *difficile* selective agar plates (CDSA, Becton Dickinson, Franklin Lakes, NJ) supplemented with sodium taurocholate (Pfaltz & Bauer Inc., Waterbury, CT) added at a concentration of 0.1% w/v (CDSA-T) were used for *in vitro* culture. *Escherichia coli* strain BLR(DE3) was obtained from EMD Millipore (Billerica, MA) and was used to express recombinant binary toxin used in the assays.

The Vero cell line (ATCC) was cultured according to established methodologies. Briefly, Vero cells were cultured in Eagle's minimum essential medium (ATCC) supplemented with 10% heat-inactivated fetal bovine sera (HyClone, Logan, Utah) and 100 units/mL of penicillin-streptomycin (Invitrogen, Carlsbad, CA). Insect cell lines Sf21(Kemp Biotechnology, Frederick, MD), or *expres*SF+® (Protein Sciences Corporation, Meriden, CT) were used for expression of vaccine antigens, and were maintained as suspension cultures at 27°C using Sf900-III culture medium (Invitrogen).

### Recombinant expression and purification of vaccine components using the insect cell -baculovirus system

A list of all proteins described in this manuscript can be found in Table 1. The genes encoding the clostridial toxins (5mTcdA, 5mTcdB, 4mCDTa, proCDTb) were codon optimized for expression in insect cells and cloned into a pFastBac1 transfer vector. The TcdA mutants were designed using sequence from strain VPI10463 (GenBank: AGG91568). The TcdB and binary toxin components were generated from the sequence from NAP1 strain R20291 (GenBank: CBE02479, CBE05856 and CBE05858). Recombinant baculovirus encoding the various clostridial toxins were generated using a Bac-to-Bac Baculovirus Expression System (Invitrogen).

**Table 1:** Recombinant toxins and vaccines used in this study.

Category	Name	Expression system	Description
**Recombinant binary toxin**	1mCDTa_ec	*E*. *coli*	6x Histidine tagged CDTa C3A. Expressed without a signal sequence.
	proCDTb_ec	*E*. *coli*	GST- CDTb fusion protein. Expressed with the activation domain and without a signal sequence.
	chymoCDTb	*E*. *coli*	Chymotrypsin digested proCDTb_ec
	proCDTb	Baculovirus	CDTb expressed with the activation domain and without a signal sequence.
	4mCDTa	Baculovirus	CDTa C3A, S346F, E386Q, E388Q. Expressed without a signal sequence.
	3mCDTa	Baculovirus	CDTa S346F, E386Q, E388Q. Expressed without a signal sequence.
**Recombinant large clostridial toxins**	4mTcdA	Baculovirus	TcdA W101A, D287A, E514Q, W519A
	5mTcdA	Baculovirus	TcdA W101A, D287A, E514Q, W519A, C700A
	4mTcdB	Baculovirus	TcdB W102A, D288A, E515Q, W520A
	5mTcdB	Baculovirus	TcdB W102A, D288A, E515Q, W520A, C698A
**Recombinant toxoids**	5mTxdA	Baculovirus	Formalin inactivated 5mTcdA
	5mTxdB	Baculovirus	Formalin inactivated 5mTcdA
**Vaccine formulations**	Bivalent	Baculovirus	5mTxdA and 5mTxdB formulated with either ISCOMATRIX™ or ISCOMATRIX™-AAHS
	Tetravalent	Baculovirus	5mTxdA, 5mTxdB, 4mCDTa and proCDTb formulated with either ISCOMATRIX™ or ISCOMATRIX™-AAHS
	Binary toxin	Baculovirus	4mCDTa and proCDTb formulated with ISCOMATRIX™

For recombinant protein generation, insect cells were grown in an Erlenmeyer flask or 20-L Wave® bioreactor (GE Healthcare, Pittsburgh, PA). The cultures were grown to a cell concentration of 1–2 x 10^6^ cells/mL and infected at an MOI of 0.1–1.0. Cells were harvested after 4–5 days of infection. The insect cells were lysed using a non-ionic detergent and clarified by centrifugation. The initial capture step for all toxins involved ion exchange chromatography followed by purification using a combination of chromatographic techniques (hydroxyapatite, multi-modal, and hydrophobic interaction) yielding products that were greater than 95% pure as determined by reducing SDS-PAGE analysis. The purified antigens were concentrated and buffer-exchanged using tangential flow filtration (TFF) and stored at -70°C.

Proteins were subjected to SDS-PAGE analysis. Three micrograms purified protein were denatured for 10 minutes at 70°C and then loaded on 8–16% Tris-glycine gels (Invitrogen, Carlsbad, CA). Electrophoresis was performed under denaturing conditions and gels were stained overnight using Gelcode® Blue Stain from Thermo Fisher Scientific (Waltham, MA). The next day gels were destained in water and fixed with 12% TCA for two hours.

### Recombinant expression and purification of active binary toxin using *E*. *coli*

For use in the binary toxin cell-based neutralization assay and to assess the toxicity of binary toxin, binary toxin components were also expressed in *E*. *coli*. The genes for 1mCDTa_ec and proCDTb_ec were codon optimized for expression in *E*. *coli* (Genscript, Piscataway, NJ) and were expressed without signal peptides (Table 1). A single mutation was added to the CDTa sequence, C3A, to prevent aggregation. 1mCDTa_ec was cloned into the *E*. *coli* expression vector pET-30a (EMD Millipore) and expressed with a C-terminal 6xHis tag. proCDTb_ec was expressed as a fusion protein with a N-terminal Glutathione-S-Transferase (GST) tag using the vector pGEX-6p1 (GE Healthcare). The vectors were sequence verified and transformed into the *E*. *coli* strain BLR(DE3).

Recombinant his-tagged 1mCdtA_ec was purified from *E*. *coli* lysates via immobilized metal affinity chromatography (IMAC). Clarified lysates were loaded on a POROS 20 MC column (Applied Biosystems, Foster City, CA) pre-loaded with Ni^2+^, and following column washing, the protein was recovered by step elution with 300 mM imidazole. The IMAC product was dialyzed (50 mM MOPS pH 6.0, 50 mM NaCl) and further purified using POROS 50 HS (Applied Biosystems) cation exchange chromatography. The final product was concentrated and exchanged into the formulation buffer (50 mM HEPES pH 7.5, 150 mM NaCl) by TFF.

Recombinant GST tagged proCDTb-ec was purified from clarified *E*. *coli* lysates using step elution from a Glutathione Sepharose 4B column (GE Healthcare) according to the manufacturer’s instructions. The GST Product was polished by size exclusion chromatography (SEC) with a Superdex200 column (GE Healthcare) in SEC buffer (50 mM HEPES pH 6.5, 150 mM NaCl).

In order to reconstitute functional binary toxin, the GST tag and activation domain from proCDTb_ec were removed by proteolytic cleavage with chymotrypsin. Activated proCDTb_ec is referred to as chymoCDTb. 1mCDTa_ec and chymoCDTb were mixed at a 1:7 molar ratio to form active binary toxin.

### Formaldehyde inactivation of native and mutant large clostridial toxins

The mutant toxins, 5mTcdA and 5mTcdB, were separately treated with formalin at room temperature, dialyzed, and sterilized by filtration. Native TcdA and TcdB antigens were separately formalin treated at 2–8°C in a manner similar to that used to produce Sanofi Pasteur’s *C*. *difficile* vaccine, which is currently being evaluated in clinical trials [27].

### Cell-based toxicity and neutralizing antibody assays

The measurement of the cellular toxicities of recombinant TcdA/TcdB proteins and neutralizing antibody titers were performed using the Vero cell assay recently described [28]. The cytotoxicity assay was performed over four days. On day 1, 384 well plates were seeded with a cell suspension of Vero cells in EMEM media and incubated overnight in a humidified incubator with 5% CO_2_ at 37°C. The next day toxins were serially 2-fold diluted in EMEM media and applied onto cells. The cell plates were incubated for 48 hours. Cells were washed and then fixed using Cytofix/Cytoperm Plus fixation permeabilization kit (Becton, Dickinson, San Diego, CA). Next cells were washed and stained with 50 μl/well of Alexa Fluor 488 phalloidin stain (Invitrogen) at a concentration of 0.016 μM. Plates were incubated in the dark at room temperature for 1 hour, washed, and then 80 μl PBS was added to the wells. Data acquisition was performed by imaging of the monolayer using an ImageXpress Velos laser scanning cytometer (Molecular Devices, Sunnyvale, CA). The total cell surface area in each well was calculated by the use of cytometer software. For data analysis, the total cell surface area in each well was plotted against the toxin concentration in each dilution. The potency of a test toxin (TC50 [50% toxic concentration]) was the toxin concentration that caused 50% cytotoxicity in the well. The TC50 value was calculated by four-parameter logistic regression of the titration curve using GraphPad Prism software.

The cell based neutralizing antibody assay was performed in a manner similar to the cytotoxicity assay with the following exceptions. On the second day of the assay, sera were 2-fold serially diluted and added to either 4 μg/ml TcdA (ribotype 087, Native Antigen Co., Oxfordshire, United Kingdom) or 40 ng/ml TcdB (ribotype 027, J. Ballard, The University of Oklahoma Science Center, Oklahoma City, OK). The sera-toxin mixtures were incubated at 37°C for 75 minutes. The mixture was then applied onto cells grown in 384-well plates and incubated for 48 hours. The cells were fixed, permeabilized, and stained with Alexa Fluor 488 phalloidin as previously described and the cell monolayer was imaged using the ImageXpress Velos scanning cytometer. The titer of a test sample (ED50 [50% effective dose]) was the dilution at which cytotoxicity was decreased by 50%. ED50 was calculated by linearly interpolating between the consecutive dilutions whose signals bracket the midpoint signal. The midpoint signal is the average of the total cell surface area of medium-only control wells and that of the toxin-only control wells.

Measurement of cytotoxicity of binary toxin variants and neutralizing antibodies was also performed in a similar manner to that previously described [29]. proCDTb-ec was activated by chymotrypsin digestion prior to running the assay. The activated form of CDTb was referred to as chymoCDTb. The binary toxin cytotoxicity assay was performed over three days. On day 1, 384 well plates were seeded with a cell suspension of Vero cells in EMEM media and incubated overnight. The next day, the CDTa component and chymoCDTb were combined at a 1:7 molar ratio (CDTa to CDTb). Binary toxin was serially diluted 2-fold in EMEM medium, and applied to cells grown in 384-well plates. Cells were incubated for 48 hours. Staining, data acquisition, and data analysis were performed as described above. For ease of comparison, we describe the TC50 for binary toxin preparations in terms of CDTa concentrations only.

For the binary toxin neutralization assay, the procedure was performed as described above with the following exceptions. Serum samples were 2-fold serially diluted, followed by incubation with a 1:7 molar ratio of a binary toxin mixture of 1mCDTa_ec and chymoCDTb for 1 hour at 37°C. The serum-toxin mixture was then applied to cells. The plates were incubated for 24 hours. The cells were fixed, permeabilized, stained with Alexa Fluor 488 phalloidin and imaged as previously described. The ED50 of a test sample was calculated as listed above.

### ELISA assays

An ELISA assay was used to measure the serum IgG titer of immunized animals for TcdA and TcdB. Thermo Reacti-Bind (Thermo Lab Systems, Waltham, MA, USA) 96-well plates were coated with 50 ng per well TcdA (List Biological Laboratories, Campbell, CA), 25 ng per well TcdB (List Biological Laboratories, Campbell, CA), 250 ng per well 1mCDTa_ec or 250 ng per well proCDTb_ec in PBS and incubated overnight at 4°C. Plates were washed with PBS-0.05% v/v Tween 20 (PBS-T) three times, and blocked with 100μl of blocking/dilution solution (PBS containing 1% bovine serum albumin (Sigma, St Louis, MO, USA)) for 1.5 hours at room temperature. Hamster sera were serially diluted (fivefold steps) on the plates and incubated at room temperature for 1.5 hours. Plates were washed with PBS-T and incubated with goat anti-hamster IgG horse-radish peroxidase conjugated antibody (Abcam, Cambridge, MA, USA) for 1 hour at room temperature. Following an additional wash with PBS-T, 50μl of 3,3′,5,5′-tetramethylbenzidine substrate (Thermo Labsystems, Milford, MA, USA) was added and after 15 minutes, the reaction was stopped by the addition of 50μl of 0.4N sulfuric acid (Fisher Scientific, Pittsburg, PA, USA) and absorbance was measured at 450 nm.

### Ethics statement

All rodent experiments were performed in strict adherence with the Guide for the Care and Use of Laboratory Animals of the National Institute of Health and following the International Guiding Principles for Biomedical Research Involving Animals. Experiments were approved by the Institutional Animal Care and Use Committee at Merck Research Laboratories, West Point, PA.

The monkey study was also performed in strict adherence with the Guide for the Care and Use of Laboratory Animals of the National Institute of Health and following the International Guiding Principles for Biomedical Research Involving Animals. Monkeys were housed and the study was performed at the University of Louisiana Lafayette New Iberia Research Center (NIRC), New Iberia, LA. The monkey study was approved by the Institutional Animal Care and Use Committee at Merck Research Laboratories, West Point, PA and at the University of Louisiana Lafayette. Animals were fed Purina 5L2P monkey chow twice daily and their diet was supplemented with novel produce and foodstuff for enrichment. Tap water was provided *ad libitum*. Animals were housed indoors socially in side by side enrichment cages and were housed with compatible cage mates throughout the study. Environmental enrichment also included toys, hanging devices, puzzle feeding devices, and a perch. Positive human interaction was conducted daily by staff as well as the monitoring of the animals health.

### Mouse toxicity model

Toxicity of 5mTcdA, 5mTcdB, 5mTxdA, 5mTxdB, and mutated binary toxin in mice was determined using female CD1 mice, weighing approximately 20 g (Charles River Laboratories, Wilmington, MA). Six animals were included in each arm of the study. Serially diluted toxins, in a 0.5 mL volume, were injected intraperitoneally and the mice were observed at least twice daily for 72 hours. Animals were humanely euthanized by CO_2_ inhalation at the end of the study or if they were unable to reach food or water. The LD_50_ was calculated as the lowest dose of toxin that resulted in the death of 50% of the mice using logistic regression analysis.

### Syrian golden hamster protection model

Male Syrian golden hamsters were individually housed in sterilized boxes with micro-isolator lids. Hamsters were immunized with a 0.2 mL vaccine dose four times either on days 0, 21, 42, and 63 or day 0, 14, 28, and 42 by intramuscular injection into the quadriceps femoris muscle. The animals received one of three vaccine formulations (Table 1). The bivalent vaccine contained 10 μg 5mTxdA and 10 μg 5mTxdB and was adjuvanted with 20 ISCO units ISCOMATRIX^™^ (CSL Behring, King of Prussia, PA) or a combination of ISCOMATRIX^™^ (20 ISCO units) and amorphous aluminum hydroxyphosphate sulfate (AAHS, 90 μg). The tetravalent vaccine contained 10 μg 5mTxdA, 10 μg 5mTxdB, 5 μg 4mCDTa and 5 μg proCDTb and was adjuvanted with 20 ISCO units ISCOMATRIX^™^. The binary toxin vaccine contained 5 μg 4mCDTa and 5 μg proCDTb and was adjuvanted with 20 ISCO units ISCOMATRIX^™^. Control hamsters were injected with 20 ISCO units ISCOMATRIX^™^ alone or a combination of 20 ISCO units of ISCOMATRIX^™^ and 90 μg AAHS. Injection sites were observed daily for a minimum of 7 days to evaluate local reactogenicity. Blood was collected two weeks after the final immunization. Five-days prior to challenge, hamsters were administered 30 mg/kg clindamycin (Sigma Aldrich, St. Louis, MO) by oral-gavage. Food, water and bedding were autoclaved prior to clindamycin treatment of animals. Animals were challenged with *C*. *difficile* spores delivered by oral-gavage 21 days following the last immunization. Cages were changed daily following spore challenge and hamsters were monitored for signs of disease (wet tail, weight loss) and death. Protocols were in place to monitor hamsters at least twice per day with strict criteria to humanely euthanize moribund animals including animals that had weight loss greater than 30%, were cool to the touch, unable to reach food or water, and comatose. Trained veterinary associates and investigators having significant experience with the hamster challenge model determined whether hamsters were allowed to progress in the study or were humanely euthanized by CO_2_ inhalation. Necropsies confirmed that hamsters which died during the studies had *C*.*difficile* pathology.

### Immunogenicity of vaccine in non-human primates

Adult rhesus macaques (4–10 years old) were immunized intramuscularly in alternating contralateral deltoid muscles with 0.5mL vaccine doses on days 0, 7, and 30. Vaccines contained 20 μg 5mTxdA, 20 μg 5mTxdB, 5 μg 4mCDTa and 5 μg proCDTb and were formulated with 60 ISCO units of ISCOMATRIX^™^ or a combination of 60 ISCO units of ISCOMATRIX^™^ and 225 μg AAHS. Control animals were immunized with a toxoid vaccine composed of 24 μg TcdA and 16 μg TcdB (Native Antigen Co, Oxfordshire, UK) which had been formalin inactivated and adjuvanted with 278 μg Rehydragel LV aluminum hydroxide adjuvant (General Chemical, Parsippany, NJ). Monkeys were monitored daily for signs of illness or distress and were appropriately cared for by NIRC staff. Injection sites were observed 4 hours post immunization and then daily for a minimum of 7 days to evaluate local reactogenicity. Serum samples were obtained on days 0, 7, 21, and 45.

### Statistical analysis

All statistical analysis of the data was performed using GraphPad Prism software (GraphPad Prism Software Inc., La Jolla, Ca).

## Results

### Heterologous expression of vaccine components

**Fig 1:**
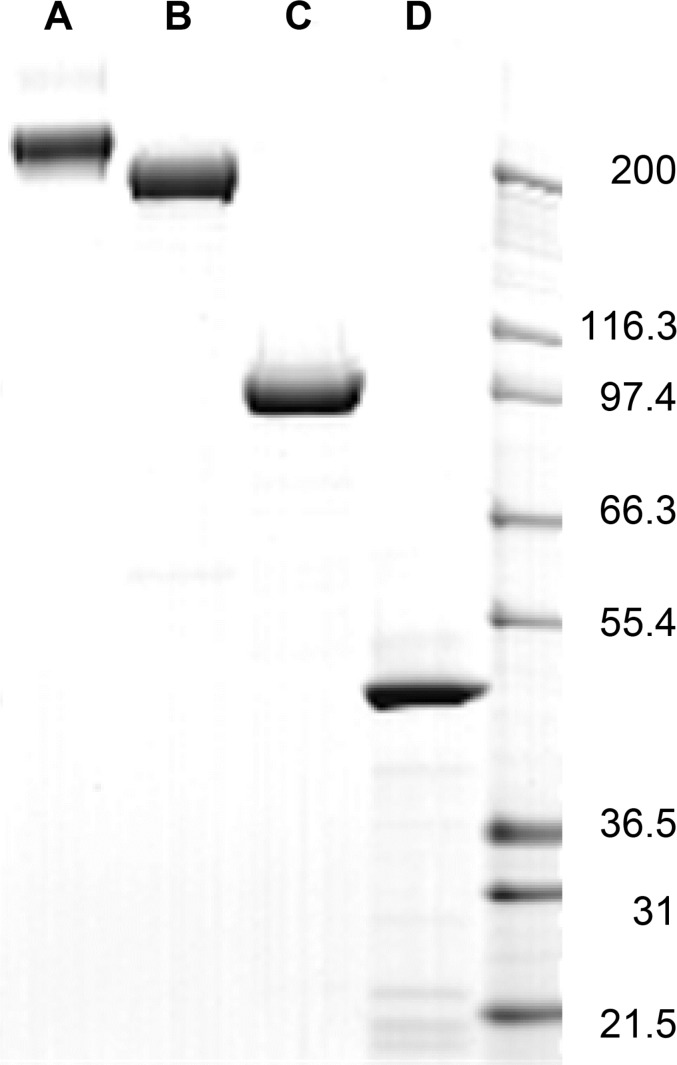
Purified vaccine components. Three micrograms 5mTcdA (lane A, 308kD), 5mTcdB (lane B, 269kD), proCDTb (lane C, 94kD) and 4mCDTa (lane D, 48kD) were loaded on a 8–16% Tris-Glycine (Invitrogen, Carlsbad, CA). GelCode Blue Stain Reagent (Thermo Fisher Scientific, Waltham, MA) was then used to stain the gel.

In order to generate the vaccine formulations, recombinant forms of TcdA and TcdB were expressed using the Bac-to-Bac® Baculovirus Expression System. The toxins were molecularly detoxified by introducing a series of four mutations in their glucosyltransferase domains (GTD). The resulting recombinant molecules, 4mTcdA and 4mTcdB, contained the amino acid substitutions described in Table 1. These mutations have been shown previously to reduce glucosyltransferase activity [30–35]. Western blot analysis indicated that 30–40% of the recombinant toxins had undergone significant proteolytic degradation (data not shown). The immune-reactive fragments corresponded in size to the toxins without the GTD suggesting that auto-proteolytic processing by the cysteine-protease domain (CPD) was occurring. Inositol hexakisphosphate (InsP6), a signaling molecule present in the cytosol of eukaryotic cells, has been shown to induce the autocatalytic cleavage of these toxins by the CPD [36]. In order to eliminate this activity and ensure product integrity, an additional mutation was added to 4mTcdA (C700A) and 4mTcdB (C698A) to generate 5mTcdA and 5mTcdB. The targeted cysteines had previously been identified as one of three active site residues in the CPD [37, 38]. Mutating these cysteine residues led to a dramatic decrease in proteolytic cleavage of the toxins resulting in less than 3% of 5mTcdA and 5mTcdB undergoing degradation (Fig 1). Using this baculovirus system we were able to produce 358 mg/L 5mTcdA and 306 mg/L 5mTcdB.

For the tetravalent vaccine, binary toxin subunits CDTa and CDTb were also recombinantly expressed using the Bac-to-Bac Baculovirus® Expression System (Table 1). To attenuate the activity of binary toxin, three mutations, which had been previously shown to reduce toxicity, were introduced in the enzymatic component to generate 3mCDTa: S346F, E386Q, E388Q [10, 39, 40]. Expression of 3mCDTa led to production of predominantly aggregated material (data not shown). As this protein contains a single cysteine at its N-terminal end, we hypothesized that the aggregation could be a result of intermolecular disulfide bond formation. To prevent aggregation, a fourth amino acid substitution was introduced replacing the cysteine with an alanine residue (C3A), generating 4mCDTa (Fig 1). Western blotting and SEC-UV analysis of 4mCDTa confirmed that this protein was >95% monomer (data not shown). CDTb was expressed in its precursor form, proCDTb. Since the pro-protein is catalytically inert, no mutations were introduced. Again protein yields were high with 245 mg/L proCDT and 587 mg/L 4mCDTa obtained.

### Genetically inactivated LCTs retained some residual toxicity

To ensure the safety of the vaccine, an *in vitro* cell-based cytotoxicity assay and an *in vivo* mouse toxicity model were used to determine if the selected mutations had fully detoxified 5mTcdA and 5mTcdB. For the cytotoxicity assay, native and mutant toxins were serially diluted and added to a monolayer of Vero cells. The potency of each toxin was determined to be the concentration that resulted in a 50% reduction in cytotoxicity to the cells (TC_50_). The results revealed that adding five mutations to these molecules greatly reduced their toxicity (Table 2) and that the toxicity was dose dependent. For 5mTcdA (TC_50_ 40 μg/mL), there was a 5.0 log reduction in toxicity compared to the native TcdA (TC_50_ 4.1x10^-4^ μg /mL) and, for 5mTcdB (TC_50_ 0.81 μg/mL), a 5.7 log reduction in toxicity was observed when compared to native TcdB (TC_50_ 1.7x10^-6^ μg /mL). Despite the fact that there was a substantial reduction in toxicity for both mutant toxins in this cellular assay, the addition of high concentrations of either toxin still resulted in substantial killing of cells.

**Table 2:** Potency of recombinant and native toxins in a cytotoxicity assay and mouse toxicity model.

	Cytotoxicity assay		Mouse toxicity model	
Toxin	TC_50_ (μg/ml)	log reduction in toxicity	LD_50_ (mg/kg)	log reduction in toxicity
native TcdA	4.1x10^-4^	0	3.2x10^-4^	0
5mTcdA	40	5.0	3.2	4.0
5mTxdA	160	5.6	> 30	>5.0
Native TcdB	1.7x10^-6^	0	2.5 x10^-3^	0
5mTcdB	0.81	5.7	0.65	2.4
5mTxdB	>540	>8.5	> 30	>7.1
1mCDTa^a^	2.4x10^-3^	0	6.5 x10^-3^	0
3mCDTa^a^	>2.0	>2.9	ND	ND
4mCDTa^a^	ND	ND	> 0.40	>1.8

^a^ CDTa components were combined with chymoCDTb at a 1:7 molar ratio (CDTa:chymoCDTb) prior to testing.

The toxicity of these molecules was also evaluated using an *in vivo* mouse toxicity model. Female CD1 mice were injected intra-peritoneally with serial dilutions of recombinant or native toxins and monitored for 72 hours. During this time, animals that were unable to reach food or water were humanely euthanized by CO_2_ inhalation. The LD_50_ was calculated as the toxin dose causing lethality in 50% of the mice (Table 2). Insertion of five amino acid changes in TcdA resulted in a 4.0 log reduction in toxicity for 5mTcdA (LD_50_ 3.2mg/kg) compared to native TcdA (LD_50_ 3.2x10^-4^ mg/kg) while a 2.4 log reduction in toxicity was observed for 5mTcdB (LD_50_0.65 mg/kg) compared to native TcdB (LD_50_2.5 x10^-3^ mg/kg). Despite the inclusion of five mutations, 5mTcdA and 5mTcdB still retained some residual toxicity, which was detectable in both the cytotoxicity assay and mouse toxicity model.

These results indicated that the mutant LCTs might require additional inactivation before they could be safely used as a vaccine. Formalin has historically been used to inactivate toxins through intramolecular and intermolecular cross-linking, while maintaining structural epitopes that are targeted by neutralizing antibodies. 5mTcdA and 5mTcdB were separately treated with formalin to generate mutant toxoids (5mTxdA and 5mTxdB) and the resulting molecules were evaluated in the *in vitro* and *in vivo* toxicity assays. Treating 5mTcdB with formalin reduced toxicity below the level of detection in both the cell-based cytotoxicity assay and the mouse toxicity model (Table 2). For 5mTxdA, all mice survived the challenge with no adverse effects even at the highest challenge dose of 30 mg/kg. However, in the cytotoxicity assay, we were able to detect a low level of toxicity for 5mTxdA (LD_50_ 160 μg/ml). Formalin treatment reduced toxicity greater than three-fold in comparison to 5mTcdA but did not completely eliminate it.

Although a low level of residual toxicity was observed for 5mTxdA in the cytotoxicity assay, we determined that 5mTxdA and 5mTxdB had been sufficiently detoxified to be safely used in animal models based on the results of the mouse toxicity model. This model revealed no adverse events in toxoid injected mice even at a dose 60 times greater than the proposed 10 μg proposed human vaccine dose. Later immunogenicity studies in hamsters and monkeys confirmed the safety of the vaccine in animals with no systemic or local adverse events observed.

### Mutations in CDTa fully inactivate binary toxin

A cell-based cytotoxicity assay, similar to the assay described above, was initially used to assess the detoxification of the molecularly attenuated binary toxin vaccine components (Table 2). Results of the cytotoxicity assay revealed that the combination of functionally active 1mCDTa_ec plus chymoCDTb induced actin depolymerization with a calculated TC_50_ of 2.4 ng/ml. The combination of 3mCDTa and chymoCDTb did not induce detectable cytotoxicity in Vero cells, suggesting that the three mutations inserted into the enzymatic region of the protein fully eliminated the toxicity of this molecule.

The mouse toxicity model detected no residual toxicity present in the binary toxin vaccine components (Table 2). CD1 mice were inoculated with binary toxin components by intraperitoneal injection and monitored for 72 hours. All of the mice survived and remained asymptomatic when challenged with 4mCDTa plus chymoCDTb, even at the highest 4mCDTa dose evaluated, 0.4 mg/kg. The combination of active 1mCDTa_ec plus chymoCDTb, yielded an LD_50_ for 1mCDTa_ec of 6.5x10^-3^ mg/kg. This suggested that the inactivating mutations had reduced toxicity at least 62-fold.

Based on the results of the cytotoxicity assay and the mouse toxicity model, we determined that the binary toxin vaccine components were fully inactivated and likely safe to use without further chemical treatment.

### Bivalent vaccine does not protect hamsters against NAP1 lethal challenge

**Fig 2:**
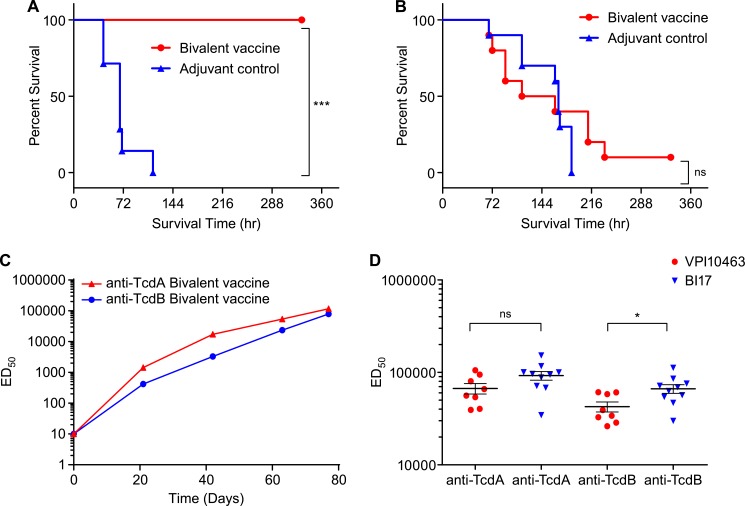
Efficacy and immunogenicity of bivalent vaccine in hamsters. Kaplan-Meier graphs show survival curves for hamsters that were immunized intramuscularly four times with the bivalent vaccine containing 10μg 5mTxdA and 10μg 5mTxdB adjuvanted with ISCOMATRIX^™^ and AAHS or adjuvant alone. Following the final immunization, hamsters were bled prior to challenge with either (A) VPI10463 (707 cfu, p<0.0001) or (B) BI17 (470 cfu, p>0.05) spores. Statistical differences in survival curves were calculated using Mantel-Cox test. (C) Pooled serum samples (day 0, 21, 42, 63 and 77) from the BI17 challenged hamsters were tested in an *in vitro* assay against Vero cells to measure neutralizing antibody activity against TcdA and TcdB. The ED50 was calculated as the serum dose that reduced cytotoxicity by 50%. Similar titers were observed from hamsters in the VPI10463 challenge. (D) Individual serum samples from day 77 from the VPI10463 and BI17 challenged hamsters were also tested for neutralizing antibody activity against TcdA and TcdB. Comparison of neutralizing antibody titers was performed using unpaired, two tailed t-test. * = p<0.05, *** = p<0.001, ns = no significant difference.

The Syrian golden hamster model is considered the gold standard for studying *C*. *difficile* pathogenesis and vaccine development. We used this model to evaluate the efficacy of the formalin-inactivated bivalent vaccine (5mTxdA and 5mTxdB) against lethal challenge with the prototypic, non-binary toxin producing strain VPI10463 as well as the epidemic, binary toxin-expressing NAP1 strain BI17. Hamsters (n = 8) were administered four intramuscular immunizations with the bivalent vaccine formulated with ISCOMATRIX^™^ and AAHS before challenge with *C*. *difficile* spores. These studies demonstrated that the bivalent vaccine was efficacious against challenge with strain VPI10463, and protected 100% of the hamsters from challenge (Fig 2A). Hamsters in the adjuvant control group began succumbing to the infection 43 hours following the challenge (n = 2) and all animals had died by the fifth day after challenge.

Unlike the results observed following challenge with VPI10463, this bivalent vaccine did not protect hamsters from a lethal challenge with strain BI17, as animals in both the vaccine and adjuvant control groups began dying by day 3 post-challenge (Fig 2B). All of the hamsters in the control group and 60% of the hamsters in the vaccine group had died by day 8, and only one hamster in the vaccine group survived for the duration of the study. In contrast to VPI10463 challenge (p<0.0001), the bivalent vaccine did not provide statistically significant protection from challenge with this NAP1 strain in comparison to the adjuvant only control animals (p>0.05).

All of the hamsters in the both studies exhibited some symptoms of CDI following challenge including weight loss and soft stools, loose stools and/or wet tail by day 5 of the challenge. At the end of the VPI10463 challenge study, one surviving hamster still had soft stool and five out of eight hamsters had begun to gain weight. The surviving hamster from the NAP1 challenge still had loose stools and continued to lose weight.

### Bivalent vaccine produced potent toxin neutralizing antibody responses in both hamster studies

To determine if the difference in hamster survival was related to immune interference between the components of the multivalent vaccine, a cell-based assay was developed to measure toxin neutralizing antibody responses in the animals that were subsequently challenged with either VPI10463 or BI17 strains. Serum was collected from hamsters prior to each immunization and 14 days after the final immunization and tested in this assay to quantitate neutralizing activity for TcdA (ribotype 087) and TcdB (ribotype 027). Neutralizing antibodies to TcdA and TcdB were generated after one immunization (Fig 2C) and continued to increase after subsequent inoculations. Hamsters from both studies generated potent neutralizing antibody titers to both toxins (Fig 2D). While there was no significant difference in TcdA neutralizing titers between the two studies (p>0.05), titers for TcdB were actually significantly higher in the animals subsequently challenged with NAP1 in comparison to animals that received the VPI10463 challenge (p<0.05). However, despite these potent antibody responses, the hamsters were not protected from NAP1 lethal challenge, indicating that neutralizing antibodies to TcdA and TcdB alone were not sufficient for protection of hamsters from NAP1 infection.

Since potent neutralizing antibody responses were generated in both studies, we theorized that the difference in survival observed was due to increased virulence of the NAP1 strain used (BI17). Most NAP1 strains produce a third toxin, binary toxin, which has been linked to increased virulence, higher disease recurrence and greater case fatality rates in a clinical setting [41–44], and therefore, we postulated that neutralizing antibodies to binary toxin, in addition to antibodies targeting the LCTs, were necessary for protection following a NAP1 challenge of hamsters.

### Inclusion of binary toxin in the vaccine significantly enhances protection from CDT+ strains

We therefore compared the protective efficacy of a tetravalent vaccine (5mTxdA, 5mTxdB, 4mCDTa, and proCDTb) against the bivalent vaccine (5mTxdA and 5mTxdB) in the Syrian golden hamster challenge model. We compared protection following challenge with a lethal dose of spores isolated from binary toxin negative strain VPI10463 (TcdA+, TcdB+,CDT-) and binary toxin producing strains BI17 (TcdA+, TcdB+, CDT+) and 8864 (TcdA-, TcdB+, CDT+).

**Fig 3:**
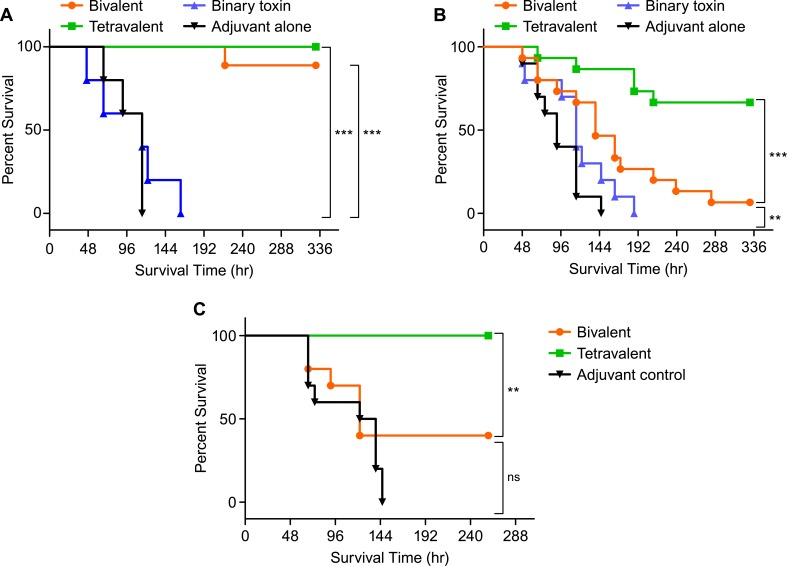
Survival curves for immunized hamsters following a lethal challenge with *C*. *difficile* VPI10463, BI17, or 8864 spores. Hamsters were immunized intramuscularly four times with the bivalent vaccine, tetravalent vaccine, binary toxin vaccine, or adjuvant alone. The 8864 challenge study did not include the binary toxin vaccine arm. (A) Hamsters (n = 9 for bivalent and tetravalent vaccine, n = 5 binary toxin vaccine or adjuvant control) were challenged with a lethal dose of VPI10463 spores (1200 cfu). (B) Hamsters (n = 15 for bivalent and tetravalent vaccine, n = 10 adjuvant control) received a lethal dose of NAP1 strain BI17 spores (425cfu). (C) Hamsters (n = 10 for all groups) received a lethal dose of strain 8864 spores (85cfu). Statistical analysis of survival curves was performed using Mantel-Cox test. * = p<0.05, ** = p<0.01, *** = p<0.001, ns = no significant difference.

For VPI10463 challenge, hamsters were immunized intramuscularly with either the bivalent vaccine or tetravalent vaccine formulated with ISCOMATRIX^™^. Since similar immunogenicity had been observed in previous studies whether the vaccine was formulated with either ISCOMATRIX^™^ alone or ISCOMATRIX^™^ plus AAHS (discussed later in this manuscript), AAHS was not included in these formulations. To determine the protective effect of binary toxin in the absence of the large clostridial toxins, a study arm was included in which hamsters were immunized with 4mCDTa plus proCDTb formulated with ISCOMATRIX^™^. Control hamsters (n = 5) were intramuscularly injected with adjuvant alone. As expected both the bivalent and tetravalent vaccines were able to provide significant protection from the VPI10463 challenge in comparison to the adjuvant alone and binary toxin alone vaccine groups (Fig 3A, p<0.001). No significant difference in survival was observed between hamsters receiving the bivalent and tetravalent vaccines (90% survival and 100% survival respectively, p>0.05). Hamsters immunized with either binary toxin alone or those in the control group died within 163 hours of the VPI10463 challenge.

In contrast to the results obtained following VPI10463 challenge, the tetravalent vaccine provided significantly better protection compared to the bivalent vaccine following challenge with binary toxin producing strains (Fig 3B and 3C). Hamsters receiving the tetravalent vaccine were significantly better protected from death from a NAP1 challenge (Fig 3B) with strain BI17 (67% survival) than those receiving the bivalent vaccine (7% survival, p<0.001). They also demonstrated significantly enhanced survival following the 8864 challenge (Fig 3C) compared to the bivalent vaccine group (100% and 60% respectively, p<0.01). All control hamsters that received the binary toxin only vaccine or ISCOMATRIX^™^ alone died by day 8. While the bivalent vaccine was able to provide protection from a VPI10463 challenge, only the tetravalent vaccine was able to protect hamsters from all three challenge strains.

Again all of the hamsters in the studies exhibited some symptoms of CDI during the course of the challenge including weight loss and soft stool, loose stool and/or wet tail by day 5 of the challenge. For the VPI10463 challenge, all of the hamsters in the bivalent vaccine group and 8 out of 9 hamsters in the tetravalent vaccine group had begun to gain weight by the end of the study. Hamsters receiving the bivalent vaccine also had normal stool by the end of the study, however, in the tetravalent vaccine group 3 out of 8 animals still exhibited wet tail. For the BI17 challenge, most of the surviving hamsters had begun to recover by the end of the study, although two hamsters that received the tetravalent vaccine continued to lose weight and had soft stool. Finally, for the 8864 challenge, all hamsters had begun to gain weight by the end of the study. Two hamsters from the bivalent vaccine group and four hamsters from the tetravalent vaccine group still had loss stool.

### Comparison of immunogenicity of bivalent and tetravalent vaccines in immunized hamsters

**Fig 4:**
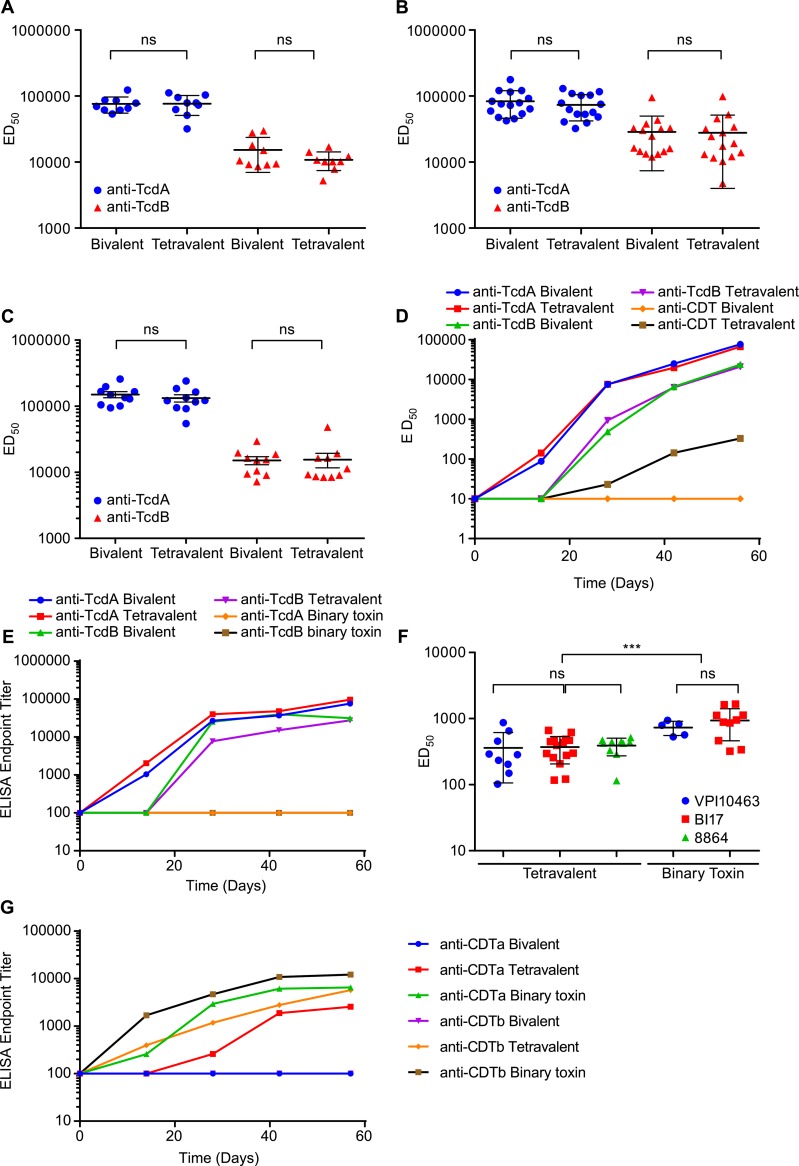
Comparison of immunogenicity of the bivalent or tetravalent vaccine in hamster vaccinated. Sera from immunized hamsters (Fig 3) were assayed for functional antibody titers to TcdA, TcdB and binary toxin in a cell based assay. The ED50 was calculated as the serum dose that reduced cytotoxicity by 50%. There was no significant difference in TcdA and TcdB neutralizing antibody titers at day 56 in (A) the VPI10463 challenge, (B) the BI17 challenge, and (C) the 8864 challenge. Data was analyzed by two tailed t-test. (D) Pooled serum samples from the BI17 challenged animals were used to measure neutralizing antibody responses to TcdA, TcdB and binary toxin at days 0, 7, 28, 42 and 56. (E) ELISA binding titers were measured in pooled sera from B17 challenged hamsters for TcdA and TcdB. (F) Strong neutralizing antibodies were also generated to binary toxin by the tetravalent and binary toxin vaccines in the VPI10463, BI17 and 8864 challenges at day 56. One way ANOVA and Tukey's multiple comparisons tests were used to compared binary toxin titers. (G) Pooled serum samples from the BI17 challenged animals were also tested for binary toxin binding antibodies. Similar kinetics were observed in the VPI10463 and 8864 studies for neutralizing and binding antibody titers. * = p<0.05, ** = p<0.01, *** = p<0.001, ns = no significant differences.

Serum neutralizing anti-toxin titers were evaluated in the cell-based assay against TcdA, TcdB and binary toxin. The bivalent and tetravalent vaccines induced similar neutralizing antibody titers to TcdA in the VPI10463 (Fig 4A), BI17 (Fig 4B), and 8864 challenged animals (Fig 4C). TcdB neutralizing titers were also comparable for the VPI10463 (Fig 4A), BI17 (Fig 4B), and 8864 (Fig 4C) challenged hamsters. Kinetics of the TcdA and TcdB neutralizing and binding antibody responses were similar whether hamsters received the tetravalent or bivalent vaccines (Fig 4D and 4E). Only hamsters receiving the tetravalent and binary toxin alone vaccines had elevated neutralizing titers to binary toxin (Fig 4D–4G). These results support our earlier finding that the increased lethality observed in the NAP1 challenge was not a result of immune interference in the tetravalent vaccine. The ability to neutralize all three toxins appears to be the sole factor differentiating between survival and death in hamsters challenged with binary toxin producing strains.

### The tetravalent vaccine generates potent neutralizing antibody titers in rhesus macaques

**Fig 5:**
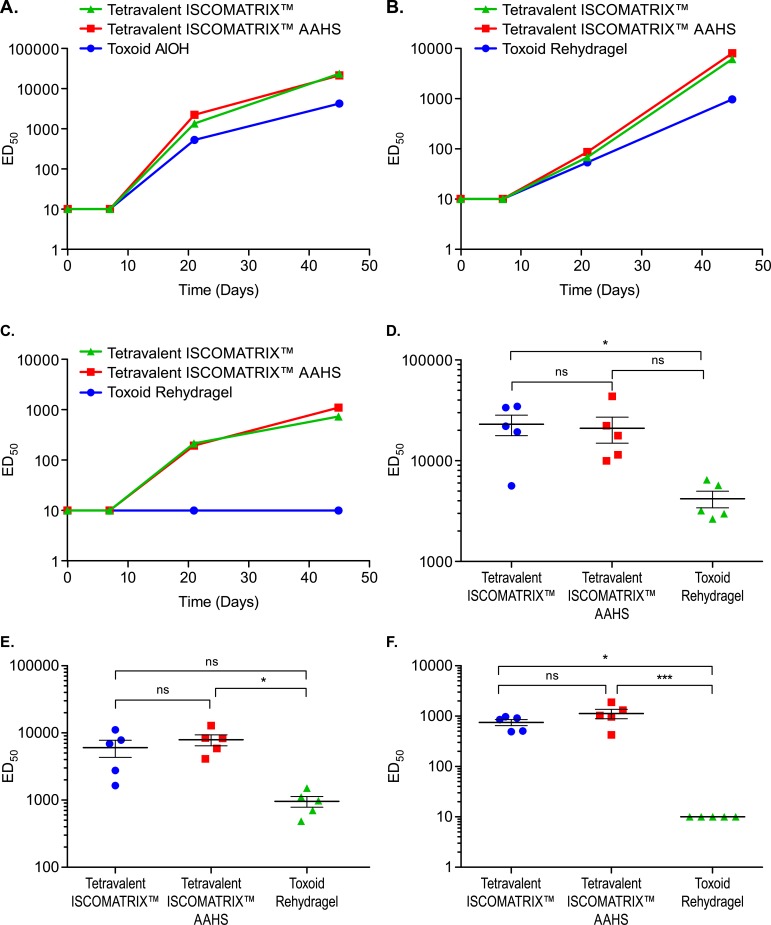
Tetravalent vaccine generates high neutralizing antibody titers in rhesus macaques. Rhesus macaques (n = 5) were given 3 immunizations with the tetravalent vaccine formulated with either ISCOMATRIX™ or ISCOMATRIX^™^ plus AAHS on d0, d7, and d30. Control animals were immunized on the same days with a formalin inactivated toxoid vaccine adjuvanted with Rehydragel. Serum samples were collected on days 0, 7, 21 and 45. Individual (Day 45) or pooled (days 0, 7 and 21) serum samples were preincubated with active toxin prior to being added to Vero cells. ED50 values indicate the serum dilution at which the area of the Vero cell monolayer is reduced by 50%. Strong neutralizing antibody titers were generated against (A) TcdA, (B) TcdB, and (C) binary toxin. Day 45 titers in these graphs represent the mean value of the individual neutralizing titers. Neutralizing antibody titers to D) TcdA, E) TcdB, and F) binary toxin were measured from individual rhesus macaque serum samples on day 45. Statistical analysis was performed using One-way ANOVA and Tukey multiple comparisons test. * = p<0.05, ns = no significant difference.

We also evaluated the immunogenicity of the tetravalent vaccine in a non-human primate immunogenicity model in order to benchmark it against a toxoid vaccine. Rhesus macaques (n = 5) were immunized intramuscularly with three doses of the tetravalent vaccine formulated with ISCOMATRIX ™ or ISCOMATRIX^™^ plus AAHS. Control monkeys received three doses of a toxoid vaccine composed of native TcdA and TcdB which had been formalin inactivated and adjuvanted with Rehydragel LV aluminum hydroxide adjuvant. None of the immunized monkeys showed any signs of reactogenicity at the injection site at any time point during this study. Two immunizations were required to detect neutralizing antibody titers for each of the three toxins and titers continued to rise after the third immunization (Fig 5A–5C). In order to assess variability between animals, neutralizing titers from individual rhesus macaques were measured at day 45 (Fig 5D–5F). Similar end point titers were observed whether the rhesus macaques received the recombinant vaccine with ISCOMATRIX^™^ or ISCOMATRIX^™^ plus AAHS (TcdA p>0.05, TcdB p>0.05, binary toxin p>0.05). Higher TcdA and TcdB neutralizing titers were induced by immunization with the tetravalent vaccine with either adjuvant when compared with the native toxoid vaccine (p<0.05).

## Discussion

Traditional vaccine development against CDI has focused on either toxoid or inactive fragments of TcdA and/or TcdB, the only known causative agents of CDI [19, 20, 22–24, 45]. Here we describe a novel tetravalent vaccine composed of inactivated TcdA, TcdB and binary toxin, expressed in insect cells using a baculovirus system. Initial attempts to express these proteins using several heterologous bacterial expression systems including *E*. *coli*, *Pseudomonas fluorescens*, and *Bacillus megaterium* yielded very low expression of proteins that were typically unstable and difficult to purify. Expression of these toxins in eukaryotic systems was deemed to be impractical, due to the conservation of the toxins’ targets in these systems and the perceived potential for cytopathic effects of the toxins on eukaryotic cells. However, despite the low levels of residual toxicity in 5mTcdA and 5mTcdB, we were able to stably express all four proteins at high levels in insect cells. This was likely because the residual toxicity detected in 5mTcdA and 5mTcdB was unrelated to these molecules’ endogenous glucosyltransferase activity. Our hypothesis was supported by the observation that we were unable to detect glucosyltransferase activity from 5mTcdA and 5mTcdB using a biochemical Rho-UDP glucosylation assay (data not shown). Furthermore, the cell intoxication resulting from the addition of 5mTcdA or 5mTcdB was morphologically different then that observed by the addition of native toxins (data not shown). Exposing Vero cells to the native toxins resulted in cell rounding of the still attached cells, presumably through actin depolymerization following glucosylation of cellular Rho-like proteins. Conversely, the mutant toxins did not cause cell rounding, but rather the cells detached from the plate and exhibited signs of lysis. Our findings were similar to those reported by Donald et al. [19], who also observed a similar cell phenotype with their inactivated toxins that was consistent with pore-induced membrane leakage, swelling and then lysis, and likely results from caspase activation [46]. It appears that insect cells are less sensitive to this alternative toxic mechanism, allowing production of mutated LCTs.

Our studies suggest that a bivalent vaccine composed of TcdA and TcdB, while very effective at preventing death in a hamster lethal challenge with the prototypic strain VPI10463, may not protect against death caused by NAP1 *C*. *difficile* strains. We hypothesized that this may be a result of the increased virulence associated with NAP1 strains, particularly their ability to express binary toxin. Therefore prevention of CDI caused by these hypervirulent strains may require antibodies that neutralize both binary toxin as well as the large clostridial toxins. Supporting this hypothesis is our observation that immunization of hamsters with a tetravalent vaccine protected animals significantly better than a similar bivalent vaccine from challenge with two binary toxin producing strains.

It should be noted that a vaccine against *C*. *difficile* toxins is designed to alleviate symptoms of the disease by generating neutralizing antibodies against the presumed causative agents of CDI. The vaccine is not intended to eliminate the bacterium itself. While most of the hamsters in the study showed signs of recovery by the end of the challenge studies, some hamsters continued to have bouts of loose stool and wet tail. This is presumably due to the fact that the *C*. *difficile* infection had not been fully resolved. Animals in the study also continued to shed spores throughout the study, so although some appeared to be healthy at the end of the study, they remained infectious.

Several clinical studies have found a correlation between binary toxin and increased virulence of certain *C*. *difficile* strains, including NAP1. A 2005 study found that binary toxin producing strains were more frequently associated with diarrhea with abdominal pain (p = 0.07) and with diarrhea being the cause of hospitalization for patients (p = 0.003) [47]. Moreover, several clinical studies of CDI report that NAP1 and other binary toxin producing strains were associated with more severe diarrhea and a higher case-fatality rate [41, 42, 44, 48]. A 2013 case study by Stewart *et al*. found that binary toxin was the single virulence factor in their study that could be independently associated with the recurrence of *C*. *difficile* colitis [43]. While there was no independent correlation between recurrence and mutations in the regulatory gene, *tcdC*, the combination of the presence of binary toxin genes and a *tcdC* mutation was associated with a 430% higher risk of recurrent CDI. These studies suggest that binary toxin is an important virulence factor in the pathogenesis of CDT+ strains. Furthermore, it is conceivable that binary toxin may act synergistically with the LCTs to increase the virulence of these strains.

It should be mentioned that not all case studies have found a correlation between binary toxin and increased virulence [49–51]. The Goldenberg and French study did, however, find a correlation between binary toxin and 30-day all-cause mortality. Discrepancies in these studies may be due to differences in the study design or size of the patient population included in the study. Additional and larger studies need to be performed to establish if there is a true correlation between binary toxin and increased virulence.

There is also anecdotal evidence that binary toxin can act as a virulence factor by itself in the absence of TcdA and TcdB. *C*. *difficile* strains which do not contain functional genes for LCTs, but do express binary toxin, have been isolated from patients in clinical settings. In one study, eight clinical isolates were identified which were A-B-CDT+, two of which had been isolated from symptomatic patients [52]. It was unclear in this study, however, if binary toxin was a virulence factor in these infections. Another study reported isolating non-toxigenic strains from symptomatic patients in a hematology and oncology ward [53]. Thirteen out of 36 *C*. *difficile* isolates recovered from patients with diarrhea, tested negative for toxins A and B by ToxA/B ELISA and PCR. The authors stated that no other intestinal pathogens were isolated from the stool samples of these patients. Of the seven patients with the highest frequency of diarrhea, non-toxigenic *C*. *difficile* were isolated in four cases. It should be noted that these isolates were not tested for the production of binary toxin. Since hospitals generally do not screen for binary toxin, it seems likely that the disease burden from non-toxigenic strains is underrepresented. These studies support the need for additional work to determine if A-B-CDT+ strains are pathogenic in a clinical setting.

In this report, we presented evidence suggesting that binary toxin is an important virulence factor in animals. Binary toxin, when injected intraperitoneally, killed both hamsters and mice in the absence of TcdA and TcdB. This finding is supported by a recently published paper by Kuehne et al [15]. The authors reported that an isogenic double mutant of NAP1 strain R20291 (A-B-CDT+) was capable of causing disease in hamsters with 3/9 hamsters dying after an oro-gastric inoculation. Taken together, these data suggest that binary toxin alone can cause disease in mammals.

There have been at least two studies where investigators have challenged non-immunized hamsters with naturally occurring strains that were of the genotype A-B-CDT+. In a study performed in our laboratory, hamsters were infected intra-gastrically with A-B-CDT+ strain IS58 (Maja Rupnik, Ljubljana, Slovenia). Five out of seven hamsters developed symptoms of CDI within 7 days of the challenge but none of the hamsters succumbed to CDI during the observation period. In addition, Geric *et al*. inoculated hamsters with four different A-B-CDT+ isolates [14]. The authors found that while some of the hamsters became colonized, none of the hamsters developed diarrhea or died and no histological changes were detected in the cecal tissues evaluated.

It is unclear why a difference in pathology was observed between the wild type A-B-CDT+ isolates and the R20291 knockout strain, however it may be due to differences in binary toxin production. In *in vitro* studies, CDT+ isolates have been found that do not produce detectable levels of either one or both binary toxin components [54, 55]. These studies have also found CDT+ strains with no detectable ADP-ribosyltransferase activity. Another possibility that cannot be discounted is that binary toxin is merely a marker for more virulent strains and that the enhanced virulence demonstrated by NAP1 isolates is due to the presence of other unknown virulence factors present in these strains. However, the data presented here supports the concept that binary toxin contributes to virulence in various animal species.

There is considerable evidence supporting binary toxin as an important virulence factor acting either independently or synergistically with the LCTs. Based on these findings, we evaluated the impact of binary toxin in the increased virulence of the NAP1 hamster challenge. To neutralize this toxin we added both components of binary toxin to our bivalent vaccine. This tetravalent vaccine continued to provide significant protection from a VPI10463 strain challenge and was also able to generate potent neutralizing antibody responses to the LCT similar to a bivalent vaccine. In addition, the tetravalent vaccine also generated potent neutralizing antibodies to binary toxin and significantly enhanced survival in hamsters from a NAP1 challenge compared to the bivalent vaccine as well as inducing potent neutralizing antibody responses to all three toxins in non-human primates. These data support further evaluation of vaccines containing binary toxin for the prevention of enhanced disease caused by epidemic strains of *C*. *difficile*.
